# Development of a co-culture system for green production of caffeic acid from sugarcane bagasse hydrolysate

**DOI:** 10.3389/fmicb.2024.1379688

**Published:** 2024-03-19

**Authors:** Xihui Wang, Cui Zhao, Xinyao Lu, Hong Zong, Bin Zhuge

**Affiliations:** ^1^The Key Laboratory of Industrial Biotechnology, Ministry of Education, School of Biotechnology, Jiangnan University, Wuxi, China; ^2^The Key Laboratory of Carbohydrate Chemistry and Biotechnology, Ministry of Education, School of Biotechnology, Jiangnan University, Wuxi, China; ^3^Research Centre of Industrial Microbiology, School of Biotechnology, Jiangnan University, Wuxi, China

**Keywords:** caffeic acid, *p*-coumaric acid, modular co-culture, shikimate, sugarcane bagasse hydrolysate

## Abstract

Caffeic acid (CA) is a phenolic acid compound widely used in pharmaceutical and food applications. However, the efficient synthesis of CA is usually limited by the resources of individual microbial platforms. Here, a cross-kingdom microbial consortium was developed to synthesize CA from sugarcane bagasse hydrolysate using *Escherichia coli* and *Candida glycerinogenes* as chassis. In the upstream *E. coli* module, shikimate accumulation was improved by intensifying the shikimate synthesis pathway and blocking shikimate metabolism to provide precursors for the downstream CA synthesis module. In the downstream *C. glycerinogenes* module, conversion of *p*-coumaric acid to CA was improved by increasing the supply of the cytoplasmic cofactor FAD(H_2_). Further, overexpression of ABC transporter-related genes promoted efflux of CA and enhanced strain resistance to CA, significantly increasing CA titer from 103.8 mg/L to 346.5 mg/L. Subsequently, optimization of the inoculation ratio of strains SA-Ec4 and CA-Cg27 in this cross-kingdom microbial consortium resulted in an increase in CA titer to 871.9 mg/L, which was 151.6% higher compared to the monoculture strain CA-Cg27. Ultimately, 2311.6 and 1943.2 mg/L of CA were obtained by optimization of the co-culture system in a 5 L bioreactor using mixed sugar and sugarcane bagasse hydrolysate, respectively, with 17.2-fold and 14.6-fold enhancement compared to the starting strain. The cross-kingdom microbial consortium developed in this study provides a reference for the production of other aromatic compounds from inexpensive raw materials.

## Introduction

Caffeic acid (CA) is a phenolic acid compound widely found in plants with pharmacological effects such as antibacterial and antiviral, cardiovascular protection, anticancer and antioxidant (Chao et al., 2010; Sato et al., 2011; Chuu et al., 2012). Currently, CA biosynthesis has been realized in a variety of microorganisms. Zhou et al. (2021) used a modified GAL regulatory system to control the expression of the pathway genes in *Saccharomyces cerevisiae* to establish a controlled and stable CA synthesis pathway, which resulted in a CA titer of 569.0 mg/L at the shake flask level. Sakae et al. (2023) obtained 6.17 g/L of CA in *Escherichia coli* at the bioreactor level by a combinatorial engineering strategy. The biosynthesis of CA is limited by a number of factors. It has been reported that less than 10% of the carbon flow during carbon metabolism enters the shikimate (SA) pathway, which does not provide sufficient precursors for CA synthesis (Liu et al., 2019; Guo et al., 2020). The biosynthesis pathway of CA is tedious, and overexpression of a large number of pathway genes would inevitably lead to microbial metabolic burdens and thus affect productivity. Moreover, other limitations exist for the synthesis of CA. For example, the conversion of *p*-coumaric acid (*p*-CA) to CA by 4-hydroxyphenylacetate 3-hydroxylase (HpaBC) requires the participation of cofactors FAD(H_2_) and NADH. However, FAD(H_2_) is synthesized in mitochondria, which results in a low concentration of FAD(H_2_) in the cytosol. Consequently, FAD(H_2_)-dependent catalytic reactions in the cytosol are relatively inefficient (Pallotta et al., 1998; Abbas and Sibirny, 2011; Chen et al., 2014a, 2022). Chen et al. (2022) systematically designed a cycling and supply system of three cofactors (FAD(H_2_), S-adenosyl-l-methion and NADPH) in *S. cerevisiae*, which significantly increased CA (5.5 g/L) and ferulic acid titer (3.8 g/L). Secondly, the antimicrobial nature of CA is also one of the non-negligible limiting factors for its biosynthesis (Bouarab-Chibane et al., 2019; Khomsi et al., 2022).

In recent years, the rapid advances in synthetic biology and metabolic engineering have paved the way for the production of high-value chemicals in heterologous biological systems, where microorganisms can synthesize natural products using simple carbon sources (Zhang and Wang, 2016; Jiang et al., 2019; Zhao et al., 2022). Co-culture engineering provides a powerful method for expanding microbial synthesis capabilities by leveraging the unique functionality of different microbial platforms and reducing the metabolic burden by distributing complex biosynthetic pathways across different strains (Johnston et al., 2020; He et al., 2023). The advantages and capabilities of different strains are fully utilized to improve the economics of the target product (Liu et al., 2018; Yan et al., 2022; Peng et al., 2023). Recently, several co-culture systems embracing different genetically engineered microorganisms have been reported for the production of high value-added chemicals. Liu et al. (2022) designed a *S. cerevisiae*-*E. coli* co-culture system for the synthesis of hydroxytyrosol, in which *S. cerevisiae* was engineered to synthesize tyrosol, the precursor of hydroxytyrosol. Yuan et al. (2020) designed an *E. coli*-*S. cerevisiae* co-culture system for resveratrol synthesis, in which the *E. coli* in the upstream module provided *p*-CA for the downstream module, which increased the resveratrol yield.

In this work, we developed a unique consortium, *E. coli*-*C. glycerinogenes*, for robust and sustainable production of CA from sugarcane bagasse hydrolysate using two microorganisms encompassing different metabolisms. The *E. coli* that overproduced shikimate (SA) was used as the upstream module to provide the precursor SA for the downstream CA synthesis module. In the downstream module, CA was synthesized using engineered *C. glycerinogenes* capable of metabolizing xylose, and the titer of CA was elevated by cofactor engineering and transporter engineering. The co-culture system developed in this work provides a strategy for the production of CA from mixed sugar and sugarcane bagasse hydrolysate, demonstrating the potential for the production of high value-added products from low-cost feedstocks.

## Materials and methods

### Strain and culture conditions

*C. glycerinogenes* WL2002-5 (CCTCC M93018) was used as the background strain for genetic manipulation. All the strains used in this study are listed in Supplementary Table S1. The engineered yeasts were cultivated in YPD medium (peptone 20 g/L, yeast extract 10 g/L, and glucose 20 g/L, pH 6.0) or YPDX medium (peptone 20 g/L, yeast extract 10 g/L, glucose 15 g/L, and xylose 5 g/L, pH 6.0). Engineered yeast transformants were screened on YNB solid medium (yeast nitrogen base without amino acid 6.7 g/L, glucose 20 g/L, agar powder 20 g/L, pH 6.0, supplemented with histidine 100 mg/L, phenylalanine100 mg/L, or lysine100 mg/L). The recombinant *E. coli* were cultivated in YPDX medium. *E. coli* JM109 containing the recombinant plasmid was screened using LB solid medium (peptone 10 g/L, yeast extract 5 g/L, NaCl 10 g/L, and agar powder 20 g/L, pH 7.0) supplemented with ampicillin (100 mg/L). Yeast extract, peptone, NaCl, YNB, histidine, phenylalanine, and lysine were purchased from Coolaber (Beijing, China). Glucose and xylose were purchased from Macklin (Shanghai, China).

### Construction of integrative plasmids and strains

**Figure 1:**
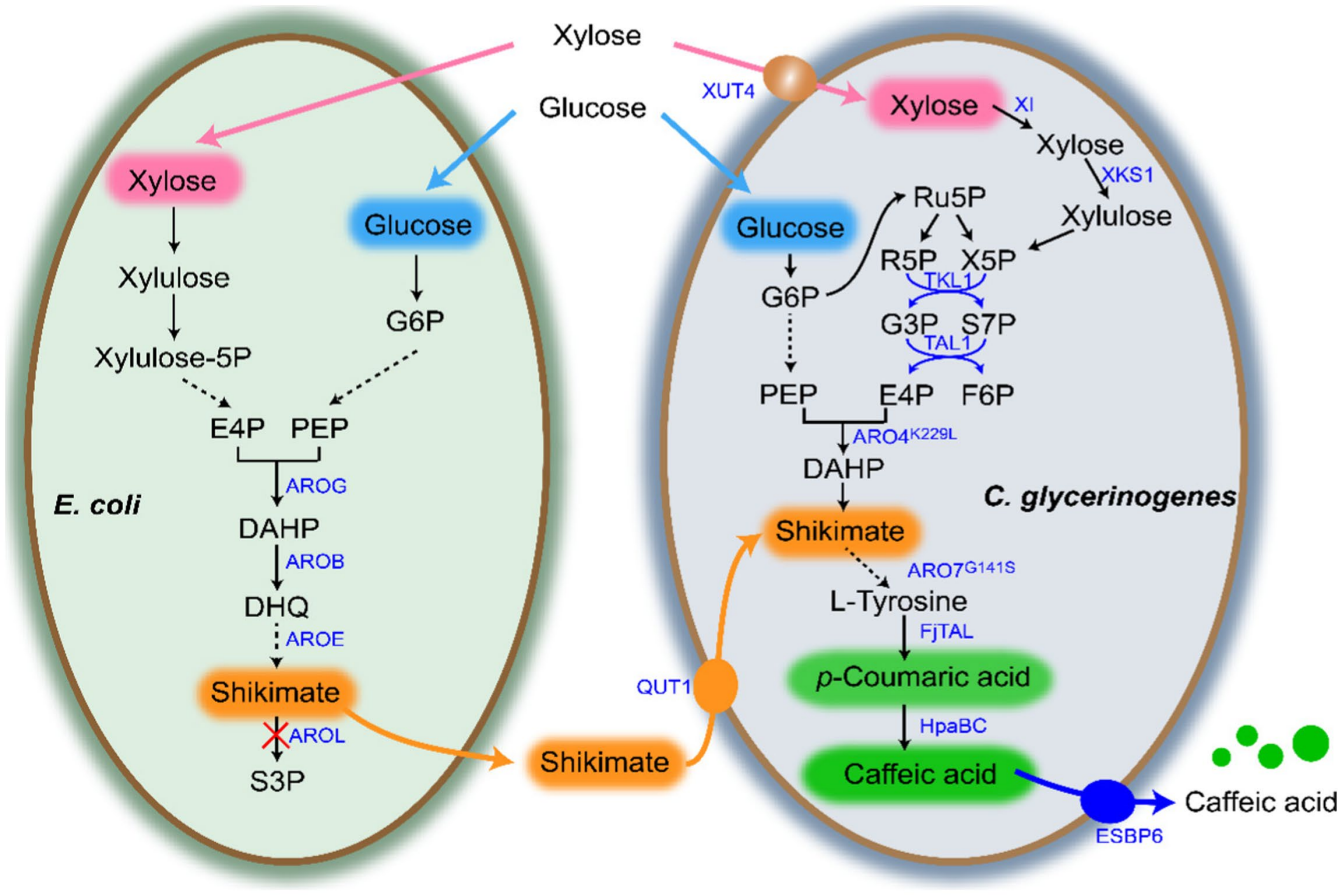
*Escherichia coli*-*Candida glycerinogenes* co-culture system for biosynthesis of CA. Gene and protein annotations: AROG, 3-deoxy-7-phosphoheptulonate synthase; AROB, 3-dehydroquinate synthase; AROE, shikimate dehydrogenase; AROL, shikimate kinase 2; XUT4, xylose transporter; XI, xylose isomerase; XKS1, xylulose kinase; TKL1, transketolase; TAL1, transaldolase; ScARO4*^K229L^*, feedback-insensitive DAHP synthases; ScARO7*^G141S^*, feedback-insensitive chorismate mutase; FjTAL, tyrosine ammonia-lyase; HpaBC, 4-hydroxyphenylacetate 3-hydroxylase. Metabolite abbreviations: DHQ, 3-dehydroquinic acid; S3P, shikmate-3-phosphate; SA, shikimate; CA, caffeic acid; *p*-CA, *p*-coumaric acid.

Supplementary Tables S1–S3 list all strains, plasmids and primers used in this study. DNA manipulation followed the methods described previously (Wang et al., 2022). Figure 1 shows the co-culture system developed in this study. In the gene overexpression plasmid construction, the plasmid pGAP was first linearized using the restriction endonuclease *BamH*I, and then the target gene was amplified and ligated to the linearized plasmid pGAP using the Assembly kit (Vazyme, China) by homologous recombination (pGAP-X). All the plasmids were verified by sequencing by Tianlin Biotechnology Co., Ltd.

For gene editing, gene knock-out or knock-in was achieved using transient CRISPR-Cas9 methods (Zhu et al., 2019). The Donor DNA, sgRNA and CgCas9 cassettes were co-transformed into auxotrophic *C. glycerinogenes* using the LiAC/SS carrying DNA/PEG method (Wang et al., 2020, 2022). All recombinant plasmids were verified by sequencing before yeast transformation. *C. glycerinogenes* transformants were screened on YNB plates supplemented with the appropriate amino acids and verified by PCR amplification from genomic DNA.

### Preparation of sugarcane bagasse hydrolysate

Sugarcane bagasse was washed, dried and crushed with a crusher. Sixty mesh sieved (diameter < 0.9 mm) and placed in a plastic bag for spare. Sugarcane bagasse hydrolysate was prepared by pretreatment with alkali (NaOH) at a concentration of 4%, with a material-liquid ratio of 1:10 (w/v), and stirred at 80°C for 2 h using a thermostatic water bath equipped with a stirrer (North-South Instruments Co., Ltd., China). At the end of pretreatment, the pH was adjusted to 9.0 with dilute H_2_SO_4_, washed until the pH was close to 7.0, then filtered and dried at 105°C. Dried bagasse was mixed with 0.05 mol/L citrate buffer in the ratio of 1:20, and cellulase (Sunson, Beijing) with 20 FPU/g was added for enzymatic hydrolysis at 50°C and 150 rpm for 48 h. The crude hydrolysate was centrifuged at 4°C and 10,000 × g for 10 min. The compositions of the final hydrolysate are shown in Supplementary Table S4, where the concentrations of glucose and xylose were at 42.4 and 15.2 g/L, respectively.

### Culture and fermentation conditions

To prepare the primary seed solution, a single colony of the engineered strain SA-Ec4 was inoculated into 10 mL of LB liquid medium and incubated at 37°C and 200 rpm for 14–16 h. Similarly, a single colony of engineered strain CA-Cg27 was inoculated into 10 mL YPD liquid medium and incubated at 30°C and 200 rpm for 14–16 h.

For shake flask fermentation, the primary seed solution was inoculated into 250 mL shake flasks containing 50 mL of YPDX medium to an OD_600_ of 1.0 and incubated at 37°C and 200 rpm, then IPTG was added at 12 h and incubated at a lower temperature of 30°C. Samples were collected at 12 h intervals for OD_600_ and yield determination. All shake flask fermentation experiments were performed in three parallel experimental groups.

To evaluate the feasibility of sugarcane bagasse hydrolysate for CA production, we carried out scale-up cultures in a 5 L bioreactor. The primary seed solutions (SA-Ec4 and CA-Cg27) prepared as described above, were inoculated into 500 mL shake flasks containing 100 mL of LB/YPD liquid medium and incubated for 14–16 h at 37°C/30°C and 200 rpm, respectively, to obtain secondary seed solutions. The secondary seed solution obtained above was inoculated in a 5 L bioreactor to an OD_600_ of 1.0 and cultivated at 37°C. IPTG was added after 12 h of fermentation and the temperature was lowered to 30°C. The pH was maintained at 6.0 by the addition of HCl (2 M) and NaOH (2 M). The rotational speed was set to 450 rpm and the air flow rate was set to 2 vvm.

### Analysis of CA, *p*-CA, glucose, xylose, SA and OD_600_

To ascertain the concentrations of CA and *p*-CA, the cultured samples were subjected to centrifugation at a speed of 10,000 × g for a duration of 10 min. The resulting supernatant was subsequently filtered using a 0.22 μm syringe filter and subjected to analysis. The quantification of CA and *p*-CA were carried out on an HPLC column (Hanbon Sci. & Tech) containing an inertsil ODS-3/C18 column (250 mm × 4.6 mm, 5 μm) with a UV detector at 310 nm. The elution method used was consistent with that described in our previous publication (Wang et al., 2022). CA yield (mg/g) = CA titer (mg/L)/ carbon source (g/L).

The concentrations of glucose, xylose and SA were determined through the application of HPLC, employing a refractive index detector (Shodex; Japan) and an Aminex HPX-87H column (300 × 7.8 mm; Bio-Rad; United States) maintained at a temperature of 60°C. The mobile phase consisted of 5.0 mM H_2_SO_4_ at a flow rate of 0.6 mL/min. An injection volume of 20 μL was employed.

In order to assess cell growth, the OD_600_ value was measured using a UV/vis spectrophotometer (Jinhua 752, Shanghai, China).

### Statistics

The assay values represent the average of three independent experiments, and the error bars represent standard errors. Statistical analysis was carried out by using student’s *t*-test (one-tailed; two-sample unequal variance; *p* = not significant (ns), ^*^p < 0.05, ^**^p < 0.01, and ^***^p < 0.001).

## Results

### Engineering *Candida glycerinogenes* for production of CA from mixed sugar

**Figure 2:**
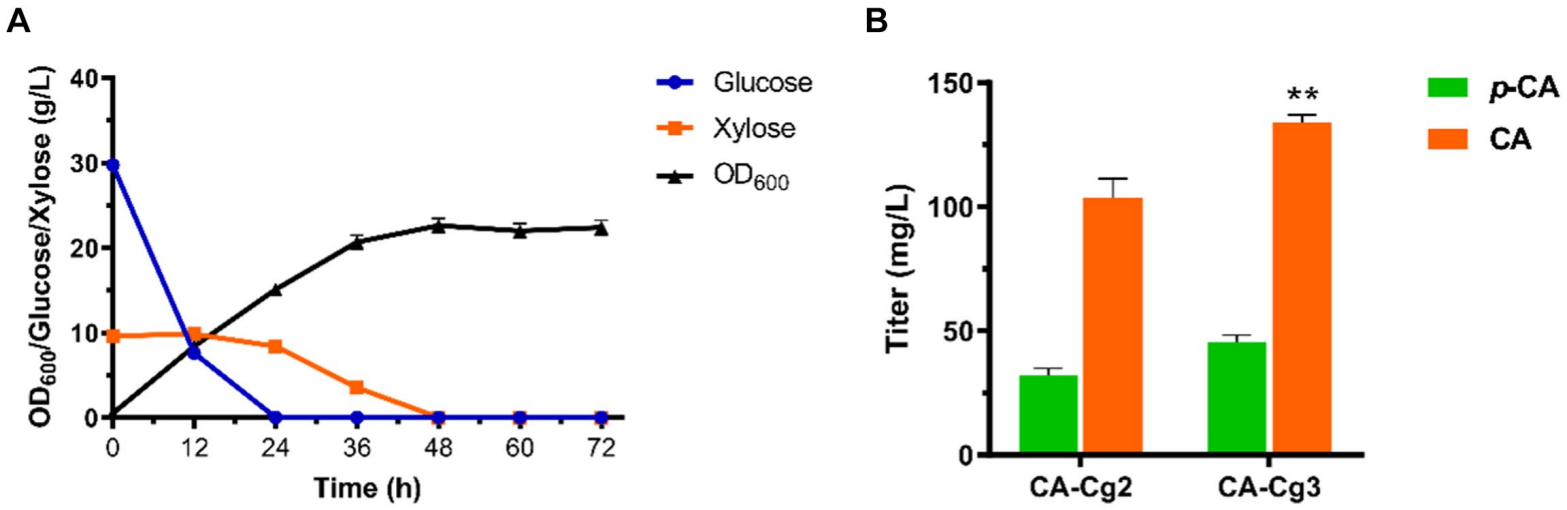
Construction of CA-producing strains. **(A)** OD_600_, glucose and xylose consumption profiles of engineered strain CA-Cg2 in YPDX medium. **(B)** CA and *p*-CA titers of strains CA-Cg2 and CA-Cg3.

In a previous study, we demonstrated the superiority of utilizing glucose and xylose fermentation for CA production in *C. glycerinogenes* (Wang et al., 2022). Therefore, in this study we first constructed the engineered strain Cg1 capable of metabolizing both glucose and xylose by introducing the cassette *XUT4*-*XI*-*XKS1*-*TKL1*-*TAL1*. After that, the CA synthesis gene *FjTAL-HpaBC* was introduced to construct the CA producing strain CA-Cg2. As shown in Figure 2A, the engineered strain CA-Cg2 was cultured in YPDX medium and glucose was first depleted, then xylose was depleted within 48 h. The OD_600_ reached 16.0 and the CA titer was 103.8 mg/L (Figure 2B). In addition, the feedback inhibition of ARO4 and ARO7 by L-tyrosine was released by introducing the mutants *ARO4^K229L^* and *ARO7^G141S^* to obtain the engineered strain CA-Cg3 (Liu et al., 2019; Guo et al., 2020). The CA titer of the engineered strain CA-Cg3 reached 134.0 mg/L (Figure 2B).

### Cofactor engineering for the improvement of CA synthesis

**Figure 3:**
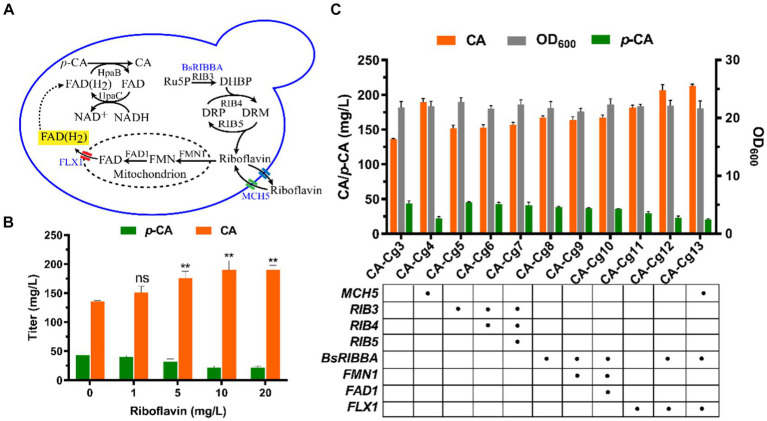
Enhancement of CA titers by modulation of cofactor FAD(H_2_) biosynthesis. **(A)** Schematic representation of FAD(H_2_) biosynthesis and transport in yeast. **(B)** CA titers of engineered strain CA-Cg3 at exogenous addition of different concentrations of riboflavin. **(C)** Effects of cofactor engineering on CA synthesis.

Riboflavin, the precursor of FAD(H_2_), is synthesized in the cytosol in a multi-step reaction, then the biosynthesis of FAD occurs in the mitochondria via FMN1 (monofunctional riboflavin kinase) and FAD1 (FAD synthase). Ultimately, FAD is transported to the cytosol via the transporter protein FLX1 to form FAD(H_2_) (Figure 3A). In order to verify the effect of cofactor FAD(H_2_) on the ability of CA synthesis, we first overexpressed the riboflavin transporter protein MCH5 in strain CA-Cg3 to obtain strain CA-Cg4. Afterwards, strain CA-Cg4 was cultured in YPDX medium supplemented with different concentrations of riboflavin. The results showed that the CA titer of strain CA-Cg4 increased with increasing riboflavin concentration and the accumulation of the intermediate product *p*-CA decreased. The titer of CA reached a maximum of 190.0 mg/L at the addition of 10 mg/L riboflavin (Figure 3B). Afterwards, we balanced the supply of cofactors by optimizing the synthetic pathways of riboflavin and FAD(H_2_). Firstly, overexpression of riboflavin synthesis pathway-related genes *RIB3*, *RIB4*, and *RIB5* alone or in combination did not achieve a significant CA titer (engineered strains CA-Cg5, CA-Cg6, and CA-Cg7) (Figure 3C). In contrast, *RIBBA* (encoding bifunctional DHBP synthase/GTP cyclohydrolase II) derived from *Bacillus subtilis* had a positive effect on promoting riboflavin synthesis, and the CA titer of the engineered strain CA-Cg8 was increased by 22.9% compared to the control strain CA-Cg3, reaching 167.1 mg/L (Figure 3C). Overexpression of the transporter protein gene *FLX1*, responsible for exporting FAD from the mitochondria to the cytoplasm, resulted in a significant increase in the CA titer of the engineered strain CA-Cg11, which was increased by 33.3% to 181.3 mg/L compared to the control strain CA-Cg3 (Figure 3C). It is worth mentioning that the accumulation of *p*-CA by strain CA-Cg11 was significantly reduced by 46.6% compared to the control strain CA-Cg3. Subsequently, the accumulation of *p*-CA was further reduced by co-overexpression of *BsRIBBA* and *FLX1*, and the CA titer of the engineered strain CA-Cg12 reached 206.4 mg/L. Furthermore, overexpression of *MCH5* in combination with the other strategies did not further enhance CA production.

### Improvement of CA production by transport engineering

**Figure 4:**
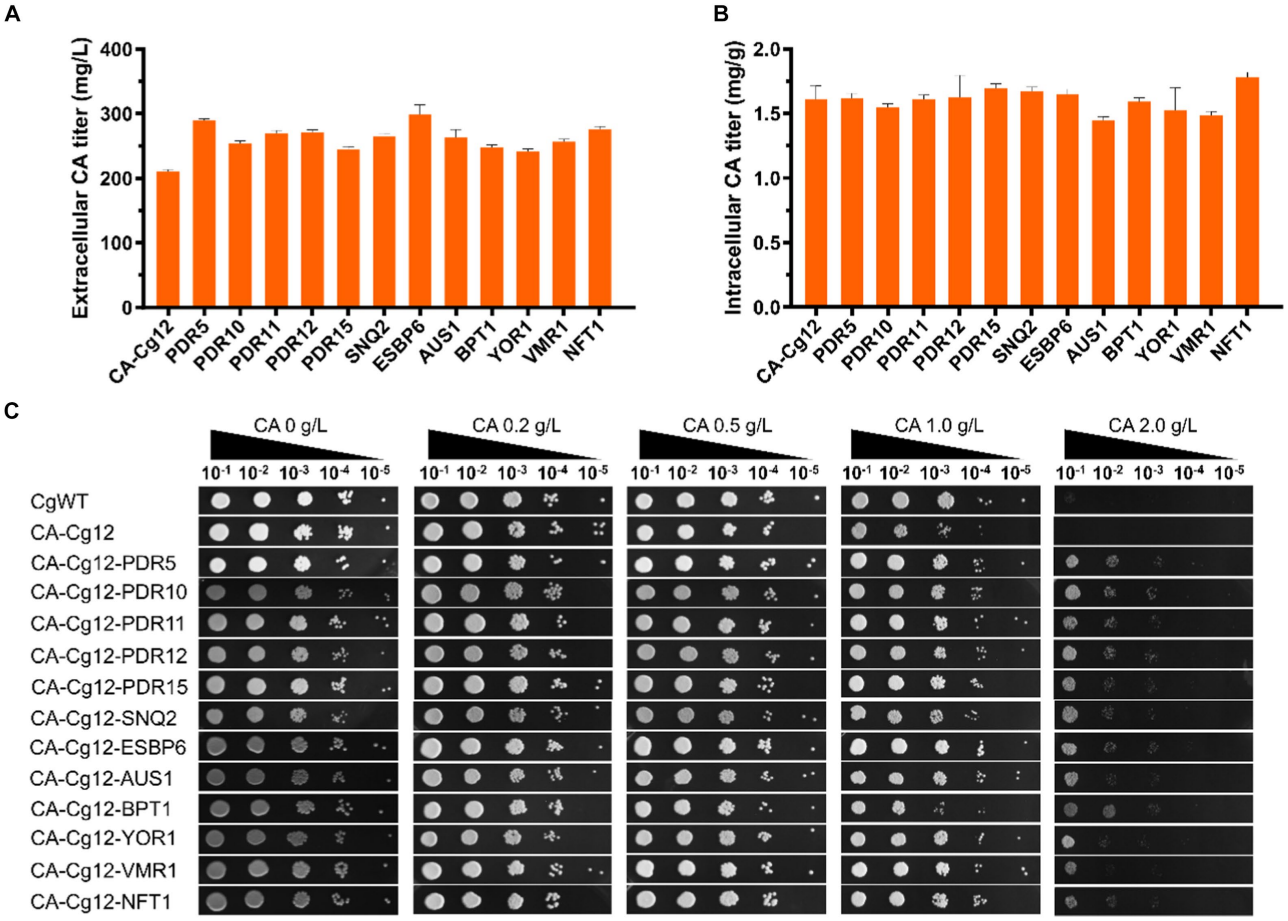
Effects of ABC transporter on CA titer. **(A,B)** Extracellular and intracellular CA titers of engineered strains overexpressing different transporter proteins. **(C)** The growth ability of the engineered strains overexpressing ABC transporter protein on YPDX medium containing different concentrations of CA. The strains CgWT and CA-Cg12 were used as negative controls and incubated at 30°C for 24 h.

We attempted to alleviate the stress of cell growth and increase extracellular CA titers by improving the efficiency of CA transport. Here, we individually expressed 12 ABC transporter proteins (PDR5, PDR10, PDR11, PDR12, PDR15, SNQ2, ESBP6, AUS1, BPT1, YOR1, VMR1, and NFT1) derived from *S. cerevisiae* in strain CA-Cg12 (Jungwirth and Kuchler, 2006; Rodriguez et al., 2017; Pereira et al., 2020; Mao et al., 2023). As shown in Figures 4A,B, all strains overexpressing the transporter proteins had higher extracellular CA titers compared to the control strain CA-Cg12, confirming that increased efflux capacity facilitates increased CA titers. Among them, strains CA-Cg14 and CA-Cg20 overexpressing the transporter proteins PDR5 and ESBP6 had the highest extracellular CA titers of 290.2 and 298.5 mg/L, which were increased by 38.1 and 42.0%, respectively, over the control strain CA-Cg12.

To verify the effect of transporter proteins on mitigating cell growth under CA stress, we performed spot plate experiments on solid YPDX medium supplemented with different concentrations of CA (0, 0.2, 0.5, 1.0, 2.0 g/L). The results are shown in Figure 4C, at CA concentrations below 1.0 g/L, the growth of the strains were almost all unaffected. At a CA concentration of 2.0 g/L, the growth of wild strain CgWT and control strain CA-Cg12 was severely inhibited and no significant colonies were found, while the strains overexpressing the transporter proteins still had the ability to grow.

### Improving CA synthesis via division of labor

**Figure 5:**
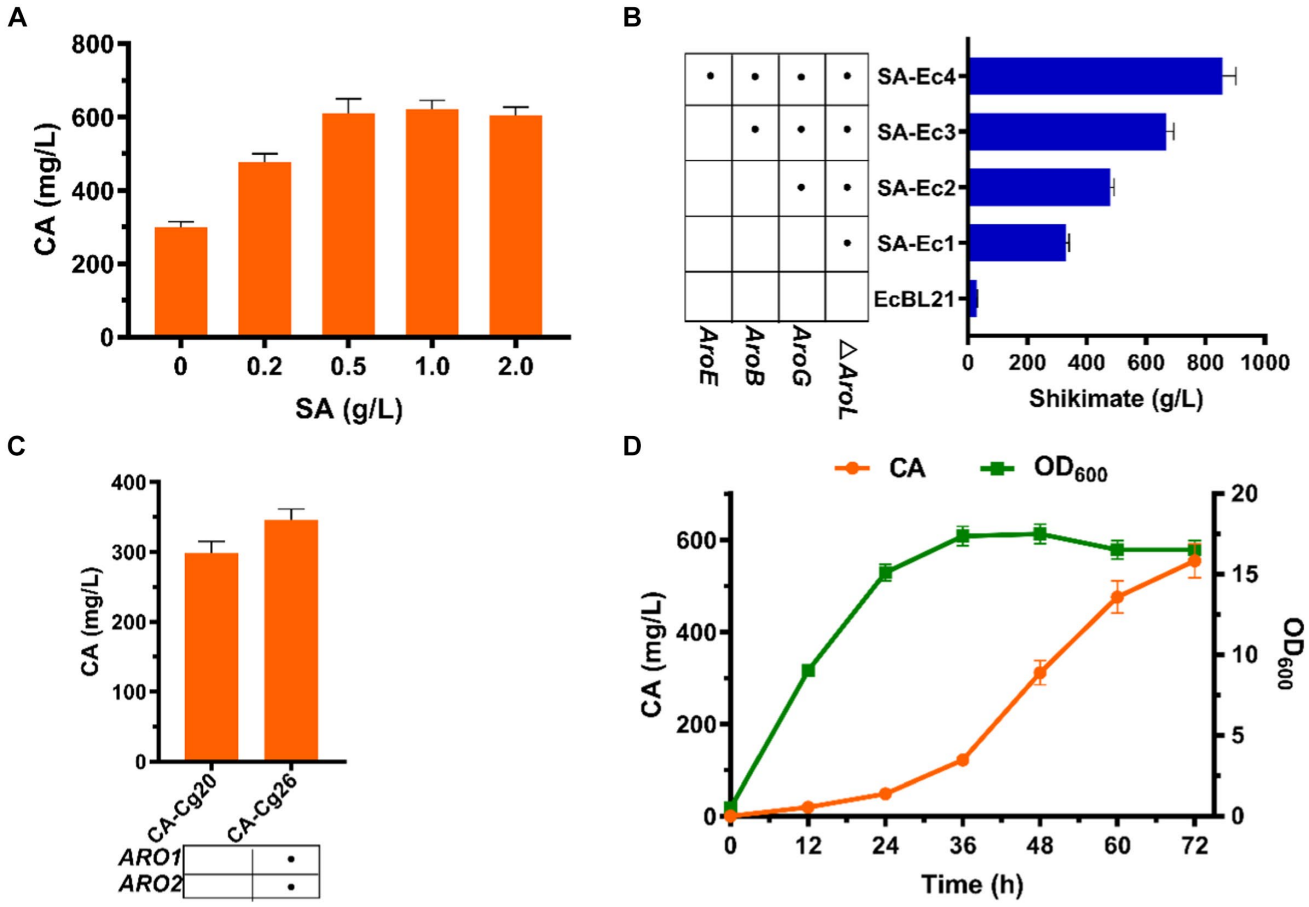
Effect of SA on CA titers. **(A)** CA titers of engineered strain CA-Cg20 at exogenous addition of different concentrations of SA. **(B)** Effect of enhanced shikimate pathway on CA titers. **(C)** Effects of metabolic pathway optimization on SA synthesis in *E. coli*. **(D)** Synthesis of CA at the shake flask level using the co-culture system (SA-Ec4: CA-Cg26).

The shikimate pathway is the only pathway for CA synthesis, and we attempted to increase the titer of CA by exogenously adding SA. As shown in Figure 5A, exogenous addition of SA had a positive effect on increasing CA titer. The CA titer was comparable when the concentration of SA was added at 0.5 and 1.0 g/L, reaching 608.9 mg/L, which was 103% improved compared to the absence of SA addition (Figure 5A). The above results indicated that SA is indeed a limiting factor for CA production. To increase the supply of SA to CA-producing strains, we enhanced the shikimate pathway by overexpressing the pathway genes *ARO1* and *ARO2* to obtain strain CA-Cg26. The results showed that after 72 h of fermentation in YPDX medium, the CA titer of strain CA-Cg26 was increased by 15.9% to 346.5 mg/L compared to the control strain CA-Cg20 (Figure 5B), indicating that simple pathway optimization was not sufficient to substantially increase the CA production.

We first selected *C. glycerinogenes* as the upstream strain of the co-culture system to produce the connecting intermediate SA using glucose and xylose to provide precursors for the downstream CA-producing strain. Therefore, we optimized the SA synthesis pathway of strain Cg1 to achieve the synthesis of SA from mixed sugars, but unfortunately, the yield of SA from strain SA-Cg4 obtained after multiple metabolic modifications remained low at 74.3 mg/L (Supplementary Figure S1). This result also indicated that the insufficient supply of SA in *C. glycerinogenes* is a key factor limiting the synthesis of aromatic compounds. Compared with *C. glycerinogenes*, *E. coli* possesses mature tools for genetic manipulation and a simple metabolic background, making it easier to obtain high yields of SA through metabolic engineering. Therefore, we chose *E. coli* as the upstream module of the co-culture system to produce SA from glucose and xylose.

To obtain a SA high yielding strain, we first blocked the SA metabolic pathway by knocking out the *AroL* gene in *E. coli* BL21 to obtain the engineered strain SA-Ec1. The results showed that the initial strain *E. coli* BL21 had a SA titer of 29.8 mg/L, and the titer of the engineered strain SA-Ec1 reached 328.5 mg/L (Figure 5C). After that, the engineered strain SA-Ec4 was obtained by stepwise overexpression of SA synthesis pathway genes *AroB*, *AroE* and anti-feedback inhibition of DAHP synthase gene *AroG^fbr^* (Chen et al., 2014b; Bo et al., 2023). As shown in Figure 5C, after 36 h of fermentation of strain SA-Ec4, the SA titer reached 857.8 mg/L, which could provide sufficient supply of precursors for CA synthesis in the downstream module of the co-culture system. Next, we tested the potential of the co-culture system comprising strains SA-Ec4 and CA-Cg26 (inoculum ratio 1:1) to improve CA synthesis. As shown in Figure 5D, this co-culture system could obtain 554.3 mg/L of CA from mixed sugar, which was 1.6 times higher than that of the monoculture strain CA-Cg26.

### Optimization of the modular co-culture system

**Figure 6:**
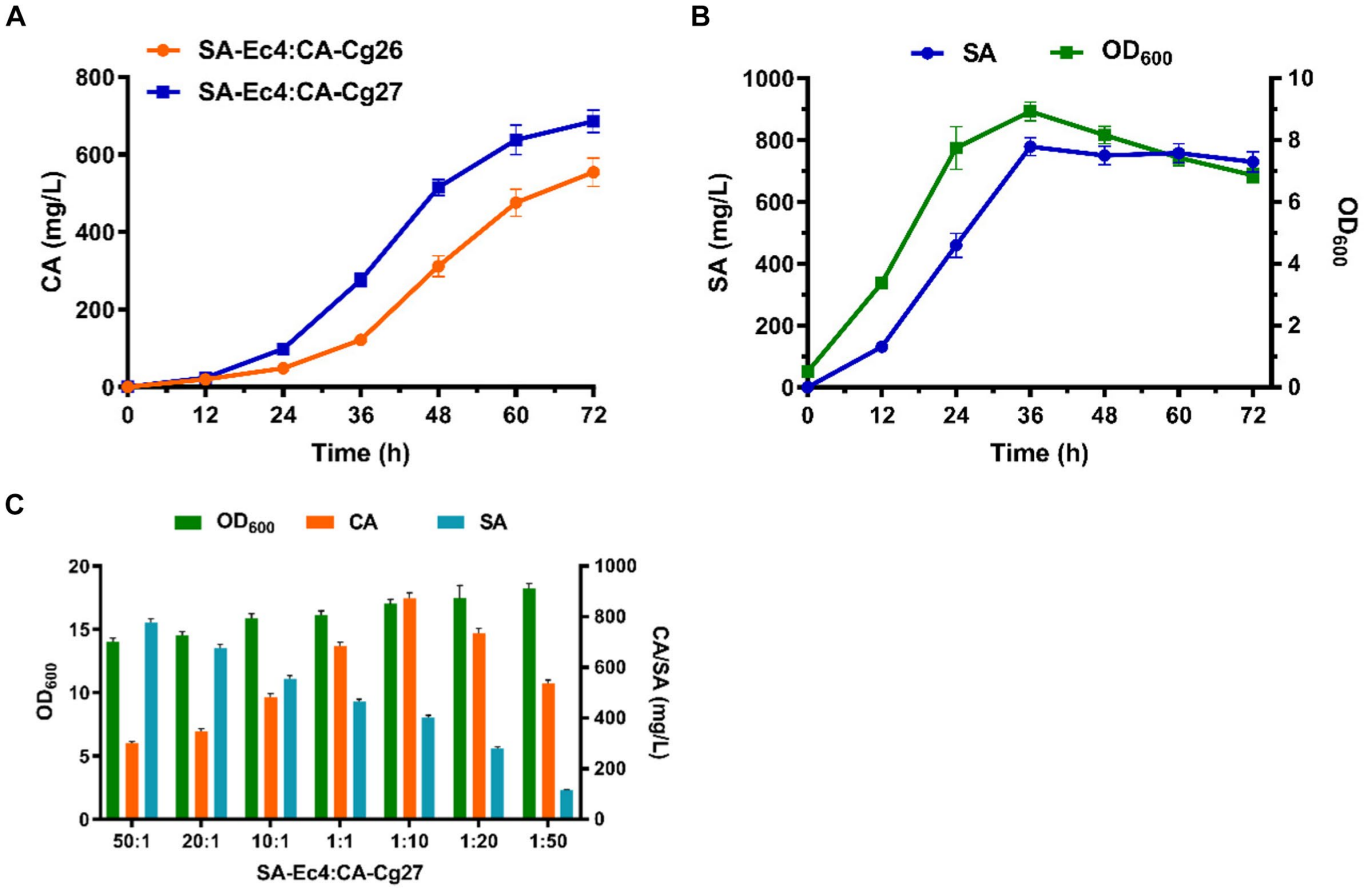
Optimization of the SA-Ec4 and CA-Cg27 co-culture system for efficient synthesis of CA. **(A)** Production of CA at the shake flask level using a co-culture system with an inoculation ratio of 1:1 (SA-Ec4: CA-Cg26; SA-Ec4: CA-Cg27). **(B)** SA production profile of engineered strain SA-Ec4 utilizing glucose and xylose. **(C)** The titers of CA and SA using co-culture systems with different inoculation ratios.

Efficient translocation of the connecting molecule is a prerequisite for building an efficient consortium. SA, as an endogenously produced metabolite, can be efficiently secreted into the medium. Therefore, increasing the uptake of SA by the CA-producing strain CA-Cg26 is the key to improving CA titers. AnQut1 (Gao et al., 2023), a quinate permease isolated from *Aspergillus niger* was introduced into strain CA-Cg26 to form strain CA-Cg27. As shown in Figure 6A, the co-culture system consisting of strain SA-Ec4 and CA-Cg27 (inoculum ratio 1:1) achieved a CA titer of 685.7 mg/L, which was 23.7% higher than that of the strain not expressing AnQut1.

Ideally, the microorganisms of modular co-culture should be interdependent to provide essential nutrients to ensure a stable and controlled microbial community (Zhou et al., 2015). Therefore, we first maintained the coexistence of strains in the microbial consortium by optimizing growth conditions. The production profile of the connecting intermediate SA in strain SA-Ec4 should be explored first, since the level of SA in the co-culture system directly determines whether CA can be robustly synthesized. As shown in Figure 6B, the SA was rapidly accumulated after 12 h and reached the highest titer at 36 h in YPDX medium for strain SA-Ec4. This indicated that the co-culture system could provide sufficient SA for the downstream CA-Cg27 at 36 h. While CA-Cg27 started to produce CA significantly at 36 h (Figure 6A). Therefore, strains SA-Ec4 and CA-Cg27 could be simultaneously inoculated into the co-culture system.

To ensure a stable and controlled microbial community, the initial inoculation ratios of SA-Ec4 and CA-Cg27 were optimized to maximize CA production at shake flask level. The medium used for the co-culture system was YPDX (15 g/L glucose, 5 g/L xylose). The initial OD_600_ of the co-culture system was 1.0, and the inoculation ratios of SA-Ec4 and CA-Cg27 were adjusted to 50:1, 20:1, 10:1, 1:1, 1:10, 1:20, and 1:50 based on OD_600_. The results showed that the CA titer was significantly affected by the inoculation ratio, i.e., the CA titer under high-ratio yeast inoculation conditions (1:50, 1:20, 1:10 for SA-Ec4: CA-Cg27) was significantly higher than that of low-ratio yeast inoculation (50:1, 20:1, 10:1 for SA-Ec4: CA-Cg27) (Figure 6C). Inoculation with high ratios of SA-Ec4 (50:1, 20:1, and 10:1 for SA-Ec4: CA-Cg27) could provide more SA for CA synthesis (Figure 6C). However, the downstream strain CA-Cg27 was unable to convert the excess SA into CA due to the relatively small percentage of CA-Cg27, resulting in wasted precursors and lower CA titers. These results emphasize that the downstream yeast strain CA-Cg27 is the critical block for CA synthesis. Among all conditions, the inoculation ratio of 1:10 (SA-Ec4: CA-Cg27) exhibited the highest CA titer of 871.9 mg/L, suggesting that the ratio of 1:10 is the optimal inoculation ratio for the co-culture module (Figure 6C).

### Production of CA in a 5 L bioreactor using a modular co-culture system

**Figure 7:**
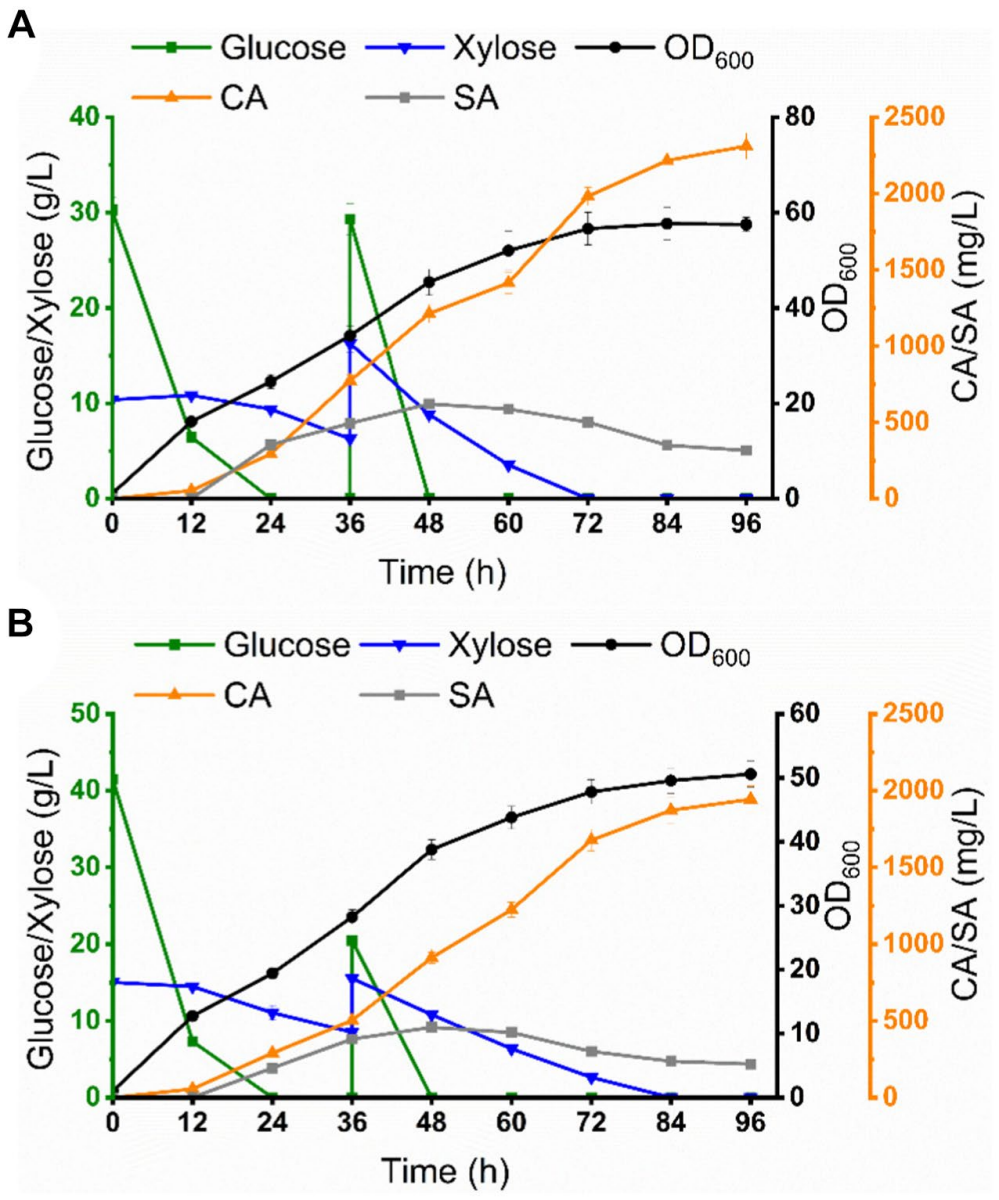
Co-culture system for CA production in a 5 L bioreactor. **(A)** Co-culture system for CA production from mixed sugar. **(B)** Co-culture system for CA production from sugarcane bagasse hydrolysate.

Scale-up cultivation is an important aspect of industrialized production. Based on the optimal conditions achieved at the shake flask level, we attempted to evaluate the scalability of this co-culture system in a 5 L bioreactor for the production of CA by fermentation utilizing mixed sugar (glucose 30 g/L, xylose 10 g/L) and sugarcane bagasse hydrolysate as carbon sources, respectively. As shown in Figure 7A, glucose was rapidly depleted during the initial phase of fermentation (0–24 h), while xylose was slowly depleted and the OD_600_ reached 47.5. The connecting intermediate, SA, began to accumulate from 24 h and reached a maximum of 621.5 mg/L at 48 h, after which SA began to fluctuate and then decline, which may be due to the fact that CA-Cg27 began to consume SA to synthesize CA. CA accumulated rapidly from 24 h and reached 2311.6 mg/L at 96 h, which was a 2.7-fold increase over the shake flask level, with a yield of 28.9 mg/g carbon source.

For CA production using sugarcane bagasse hydrolysate (glucose 42.4 g/L, xylose 15.2 g/L, the compositions of sugarcane bagasse hydrolysate were shown in Supplementary Table S4) as the carbon source, glucose was preferentially consumed, and then xylose was depleted at 72 h, and the OD_600_ reached 45.6. SA concentration reached 457.6 mg/L at 48 h and declined thereafter. CA accumulated rapidly from 24 h and reached 1943.2 mg/L at 96 h, a 2.2-fold increase over the shake flask level, with a yield of 23.4 mg/g carbon source (Figure 7B; Table 1). The titer of CA production using sugarcane bagasse hydrolysate was significantly lower than that under mixed sugar fermentation conditions, which may be attributed to the presence of inhibitory substances in sugarcane bagasse hydrolysate, such as furfural and acetic acid.

**Table 1:** Caffeic acid production in yeast and *Escherichia coli.*

Products	Host	Cultivation condition	Titers (g/L)	Yield (mg/g carbon source)	Reference
CA	*S. cerevisiae*	Shake flask, MYPD^a^	0.01	0.3	Li et al. (2020)
	*S. cerevisiae*	Shake flask, YPD	0.03	1.4	Yang et al. (2020)
	*S. cerevisiae*	Shake flask, YPD	0.57	28.5	Zhou et al. (2021)
	*S. cerevisiae*	Shake flask, YPD	1.60	80.0	Chen et al. (2022)
	*S. cerevisiae*	Fed-batch, MM^b^, glucose feeding	5.52	31.8	Chen et al. (2022)
	*C. glycerinogenes*	Shake flask, YPDX	0.87	43.6	This study
	*C. glycerinogenes*	Fed-batch, YPDX	2.31	28.9	This study
	*C. glycerinogenes*	Fed-batch, sugarcane bagasse hydrolysate	1.94	23.4	This study
	*E. coli*	Shake flask, M9Y^c^	1.58	79	Sakae et al. (2023)
	*E. coli*	5 L bioreactor	6.17	123.4	Sakae et al. (2023)
	*E. coli*	Shake flask	0.78	51.71	Wang et al. (2023)
	*E. coli*	Fed-batch, glycerol feeding	7.92	15.84	Wang et al. (2023)

a MYPD, YPD medium containing 40 g/L glucose biosynthesis efficiency under the same condition, cannot reflect the real yield from glucose since YPD containing yeast extract and peptone, which can be used for product synthesis.

b MM, minimal media.

c M9Y, the composition of the M9Y medium is detailed in reference Sakae et al. (2023).

## Discussion

In this work, we developed a unique consortium, *E. coli*-*C. glycerinogenes*, for robust and sustainable production of CA from sugarcane bagasse hydrolysate using two microorganisms encompassing different metabolisms. Ultimately, the cross-kingdom microbial consortium obtained CA titers of 2311.6 and 1943.2 mg/L in 5 L bioreactors utilizing mixed sugar and sugarcane bagasse hydrolysate with yields of 28.9 and 23.4 mg/g carbon source, respectively.

Cofactors are organic compounds required for many intracellular metabolic processes and reactions, and some key enzymes often require specific cofactors to maintain high activity. Inadequate supply of cofactors may lead to metabolic disorders during natural product biosynthesis (Chen et al., 2014b, 2022). The CA biosynthesis pathway consumes large amounts of FAD(H_2_), and thus an enhanced CA biosynthesis pathway would disrupt intracellular FAD(H_2_)/FAD homeostasis. The engineered strain CA-Cg3 successfully synthesized CA using mixed sugar, but there was still a certain amount of *p*-CA in the fermentation broth that was not completely converted to CA. It is worth mentioning that HpaBC, which is responsible for the conversion of *p*-CA to produce CA, is required for the involvement of the cofactors FAD(H_2_) and NADH. Exogenous addition of riboflavin enhances the conversion of *p*-CA to CA by providing FAD(H_2_) required by HpaB, thereby increasing the titer of CA. Overexpression of *FMN1* and *FAD1* did not positively affect CA production (strains CA-Cg9 and CA-Cg10), suggesting that *FAD1* and *FMN1* in the mitochondria are not rate-limiting steps in FAD synthesis. However, overexpression of FLX1 significantly increased CA titers, indicating that the transport of FAD from mitochondria to the cytoplasm is a critical step in limiting the availability of FAD(H_2_) in the cytoplasm. Furthermore, overexpression of *MCH5* in combination with the other strategies did not further enhance CA production, suggesting that the synthesis of FAD(H_2_), which was enhanced by the above strategies, was sufficient to supply the enzymatic reaction of HpaB during CA synthesis.

CA is usually toxic to microorganisms due to its antimicrobial action. The external presence or internal production of toxicity reduces the productivity of the microbial cell factory (Bouarab-Chibane et al., 2019; Khomsi et al., 2022). Currently, several feasible strategies have been employed to address the toxicity issue (Dunlop et al., 2011; Minty et al., 2011; Doshi et al., 2013; Zhang et al., 2016; Tan et al., 2017; Wang et al., 2017; Bu et al., 2020), among which transport engineering is considered one of the most promising strategies because it not only mitigates product toxicity but also reduces isolation costs (Lee et al., 2016; Hara et al., 2017). Among the 14 selected transporter proteins, PDR5 and ESBP6 were the most effective, and the CA titers of the engineered strains were 290.2 and 298.5 mg/L, which were 38.1 and 42.0% higher than the control strain CA-Cg12, respectively. Moreover, overexpression of the transporter protein also improved the tolerance of the strain to CA.

The shikimate pathway is the only pathway for aromatic amino acid synthesis. However, it has been estimated that less than 10% of the carbon flow enters the shikimate pathway (Liu et al., 2019; Guo et al., 2020). Therefore, we proposed that insufficient supply of SA is one of the major factors limiting CA synthesis, which was subsequently supported by the significant increase in CA titer by exogenous addition of SA. Increasing the supply of SA in CA-producing strains is the key to improving CA titers. Currently, most studies focus on metabolic modification of the upstream pathway to increase the carbon flux into the SA pathway, but cumbersome genetic modifications and lengthy metabolic pathways increase the metabolic burden of the cell. Co-culture encompassing different metabolic modules has emerged as a promising strategy, with the products of the upstream module serving as substrates for the downstream module. Dividing the target pathway into modules carried by different strains can take advantage of the individual genetic characteristics of these strains to achieve optimal yields. We performed metabolic modification of *E. coli* in the upstream module and CA-producing strains in the downstream module of the co-culture system, enabling the strain SA-Ec4 in the upstream module to provide adequate supply of precursors for the strain CA-Cg27 in the downstream module. Afterwards, by optimizing the inoculation ratio of strains SA-Ec4 and CA-Cg27 in the co-culture system, the CA titer was increased to 871.9 mg/L, which was 151.6% higher compared to the monoculture strain CA-Cg27. Ultimately, the cross-kingdom microbial consortium obtained CA titers of 2311.6 and 1943.2 mg/L in 5 L bioreactors utilizing mixed sugar and sugarcane bagasse hydrolysate with yields of 28.9 and 23.4 mg/g carbon source, respectively. The highest yields of CA production from glucose in *S. cerevisiae* and *E. coli* are currently 5.52 and 6.17 g/L, respectively. Although the highest CA yield was not achieved in this study, it is at a high level in the study of CA production using yeast cell factories. Moreover, this is the first report on the synthesis of CA using bagasse hydrolysate as a carbon source, which provides a reference for the production of CA from other inexpensive raw materials. The development of lignocellulosic hydrolysate for the production of biochemicals has a positive effect on environmental protection and food demand aspects. The co-culture system we developed has the potential to produce high value-added products from low-cost raw materials.

## Conclusion

Here, we designed and developed a cross-kingdom microbial consortium for efficient production of CA from sustainable carbon sources. Overproduction of SA by *E. coli* serves as an upstream module to provide precursors required for downstream CA synthesis. In the downstream module of *C. glycerinogenes*, the conversion of *p*-CA to CA was improved by balancing the supply of the cofactor FAD(H_2_). Further, CA titer was improved by enhanced extracellular transport. Ultimately, the cross-kingdom microbial consortium obtained CA titers of 2311.6 and 1943.2 mg/L in 5 L bioreactors utilizing mixed sugar and sugarcane bagasse hydrolysate with yields of 28.9 and 23.4 mg/g carbon source, respectively. Considering the promising titers for the synthesis of CA, the system can also be applied for the production of other high-value compounds derived from the shikimate pathway.

## Data availability statement

The original contributions presented in the study are included in the article/Supplementary material, further inquiries can be directed to the corresponding author.

## Author contributions

XW: Writing – original draft, Writing – review & editing. CZ: Methodology, Supervision, Writing – review & editing. XL: Methodology, Supervision, Writing – review & editing. HZ: Methodology, Supervision, Writing – review & editing. BZ: Conceptualization, Data curation, Methodology, Supervision, Writing – review & editing.
