# Supplementary material for: Development of a co-culture system for green production of caffeic acid from sugarcane bagasse hydrolysate
Source: Front Microbiol. 2024 Mar 19;15:1379688. doi: 10.3389/fmicb.2024.1379688 (PMC10985150; doi:10.3389/fmicb.2024.1379688)
Supplement: Supplementary file 1 [file Data_Sheet_1.PDF]

Development of a co-culture system for green production of caffeic acid from sugarcane bagasse hydrolysate

Xihui Wang[1, 2, 3], Cui Zhao[1, 2, 3], Xinyao Lu[1, 2, 3], Hong Zong[1, 2, 3], Bin Zhuge[1, 2, 3*]

[1]The Key Laboratory of Industrial Biotechnology, Ministry of Education, School of Biotechnology, Jiangnan University, 1800 Lihu Road, Wuxi, Jiangsu, 214122, China

[2]The Key Laboratory of Carbohydrate Chemistry and Biotechnology, Ministry of Education, School of Biotechnology, Jiangnan University, 1800 Lihu Road, Wuxi, Jiangsu, 214122, China

[3]Research Centre of Industrial Microbiology, School of Biotechnology, Jiangnan University, 1800 Lihu Road, Wuxi, Jiangsu, 214122, China

* **Corresponding author:** Bin Zhuge (bzhuge@126.com)

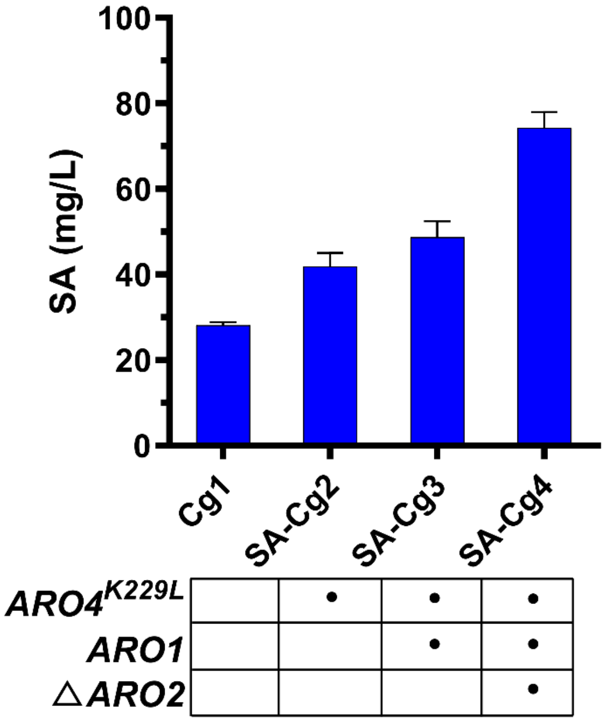

Figure S1. Effects of metabolic pathway optimization on SA synthesis in *C. glycerinogenes*.

Table S1 Strains used in this study

Strain	Relevant genotype	Reference
E. coli JM109	Wild-type	Lab stored
E. coli BL21	Wild-type	Lab stored
CgWT	*Candida glycerinogenes* WL2002-5, Wild-type	Lab stored
CgUT	*Candida glycerinogenes* URA3Δ, TRP1Δ, host for gene expression	Lab stored
SA-Ec1	*E. coli* K12; AroLΔ	This study
SA-Ec2	*E. coli* K12; deletion of *AroL* and integrated linearized pRSFDuet-*AroG* at *AroL* site	This study
SA-Ec3	*E. coli* K12; deletion of *AroL* and integrated linearized pRSFDuet-*AroG-AroB* at *AroL* site	This study
SA-Ec4	*E. coli* K12; deletion of *AroL* and integrated linearized pRSFDuet-*AroG-AroB-AroE* at *AroL* site	This study
Cg1	CgUT; deletion of *CgHIS3* and integrated linearized pGAP-URA3-*XUT4-XI-XKS1-TKL1-TAL1* at *CgHIS3* site	This study
CA-Cg2	Cg1; deletion of *CgLYS2* and integrated linearized pGAP-HIS3-*FjTAL-HpaBC* at *CgLYS2* site	This study
CA-Cg3	Cg1; deletion of *CgLYS2* and integrated linearized pGAP-HIS3-*FjTAL-HpaBC-ARO4*^{K229L}-*ARO7*^{G141S} at *CgLYS2* site	This study
CA-Cg4	CA-Cg3; deletion of *CgPHA2* and integrated linearized pGAP-TRP1-*MCH5* at *CgPHA2* site	This study
CA-Cg5	CA-Cg3; deletion of *CgPHA2* and integrated linearized pGAP-TRP1-*RIB3* at *CgPHA2* site	This study
CA-Cg6	CA-Cg3; deletion of *CgPHA2* and integrated linearized pGAP-TRP1-*RIB3-RIB4* at *CgPHA2* site	This study
CA-Cg7	CA-Cg3; deletion of *CgPHA2* and integrated linearized pGAP-TRP1-*RIB3-RIB4-RIB5* at *CgPHA2* site	This study
CA-Cg8	CA-Cg3; deletion of *CgPHA2* and integrated linearized pGAP-TRP1-*BsRIBBA* at *CgPHA2* site	This study
CA-Cg9	CA-Cg3; deletion of *CgPHA2* and integrated linearized pGAP-TRP1-*BsRIBBA-FMN1* at *CgPHA2* site	This study
CA-Cg10	CA-Cg3; deletion of *CgPHA2* and integrated linearized pGAP-TRP1-*BsRIBBA-FMN1-FAD1* at *CgPHA2* site	This study
CA-Cg11	CA-Cg3; deletion of *CgPHA2* and integrated linearized pGAP-TRP1-*FLX1* at *CgPHA2* site	This study
CA-Cg12	CA-Cg3; deletion of *CgPHA2* and integrated linearized pGAP-TRP1-*BsRIBBA-FLX1* at *CgPHA2* site	This study
CA-Cg13	CA-Cg3; deletion of *CgPHA2* and integrated linearized pGAP-TRP1-*BsRIBBA-FLX1-MCH5* at *CgPHA2* site	This study
CA-Cg14	CA-Cg3; deletion of *CgPHA2* and integrated linearized pGAP-TRP1-*BsRIBBA-FLX1-PDR5* at *CgPHA2* site	This study
CA-Cg15	CA-Cg3; deletion of *CgPHA2* and integrated linearized pGAP-TRP1-*BsRIBBA-FLX1-PDR10* at *CgPHA2* site	This study
CA-Cg16	CA-Cg3; deletion of *CgPHA2* and integrated linearized	This study

	pGAP-TRP1-*BsRIBBA-FLX1-PDR11* at *CgPHA2* site	
CA-Cg17	CA-Cg3; deletion of *CgPHA2* and integrated linearized pGAP-TRP1-*BsRIBBA-FLX1-PDR12* at *CgPHA2* site	This study
CA-Cg18	CA-Cg3; deletion of *CgPHA2* and integrated linearized pGAP-TRP1-*BsRIBBA-FLX1-PDR15* at *CgPHA2* site	This study
CA-Cg19	CA-Cg3; deletion of *CgPHA2* and integrated linearized pGAP-TRP1-*BsRIBBA-FLX1-SNQ2* at *CgPHA2* site	This study
CA-Cg20	CA-Cg3; deletion of *CgPHA2* and integrated linearized pGAP-TRP1-*BsRIBBA-FLX1-ESBP6* at *CgPHA2* site	This study
CA-Cg21	CA-Cg3; deletion of *CgPHA2* and integrated linearized pGAP-TRP1-*BsRIBBA-FLX1-AUS1* at *CgPHA2* site	This study
CA-Cg22	CA-Cg3; deletion of *CgPHA2* and integrated linearized pGAP-TRP1-*BsRIBBA-FLX1-BPT1* at *CgPHA2* site	This study
CA-Cg23	CA-Cg3; deletion of *CgPHA2* and integrated linearized pGAP-TRP1-*BsRIBBA-FLX1-YOR1* at *CgPHA2* site	This study
CA-Cg24	CA-Cg3; deletion of *CgPHA2* and integrated linearized pGAP-TRP1-*BsRIBBA-FLX1-VMR1* at *CgPHA2* site	This study
CA-Cg25	CA-Cg3; deletion of *CgPHA2* and integrated linearized pGAP-TRP1-*BsRIBBA-FLX1-NFT1* at *CgPHA2* site	This study
CA-Cg26	CA-Cg3; deletion of *CgPHA2* and integrated linearized pGAP-TRP1-*BsRIBBA-FLX1-ESBP6-ARO1-ARO2* at *CgPHA2* site	This study
CA-Cg27	CgUT; deletion of *CgHIS3* and integrated linearized pGAP-URA3-*XUT4-XI-XKS1-TKL1-TAL1* at *CgHIS3* site; deletion of *CgLYS2* and integrated linearized pGAP-HIS3-*FjTAL-HpaBC-ARO4*^{K229L}-*ARO7*^{G141S}-*QUT1* at *CgLYS2* site; deletion of *CgPHA2* and integrated linearized pGAP-TRP1-*BsRIBBA-FLX1-ESBP6-ARO1-ARO2* at *CgPHA2* site	This study
SA-Cg2	Cg1; integration of linearized pGAP-HIS3-*ARO4*^{K229L} at 5.8s rDNA site	This study
SA-Cg3	Cg1; integration of linearized pGAP-HIS3-*ARO4*^{K229L}-*ARO1* at 5.8s rDNA site	This study
SA-Cg4	Cg1; deletion of *CgARO2* and integrated linearized pGAP-HIS3-*ARO4*^{K229L}-*ARO1* at *CgARO2* site	This study

Table S2 Plasmids used in this study

**Plasmids**	**Relevant characteristics**	**Reference or Source**
pMD19-T	linearized TA-cloning vector	Lab stored
pRSFDuet	linearized TA-cloning vector	Lab stored
pCas9	P-cas9, Kan^r	Lab stored
pTargetF-PMB	sgRNA, Spe^r	Lab stored
pGAP-URA3	pMD19-T-5.8s rDNA-P_{GAP}-mcs-T_{AOX1}, URA3	Lab stored
pGAP-TRP1	pMD19-T-5.8s rDNA-P_{GAP}-mcs-T_{AOX1}, TRP1	Lab stored
pGAP-HIS3	pMD19-T-5.8s rDNA-P_{GAP}-mcs-T_{AOX1}, HIS3	This study
pMY-CgCas9	pMD19-T-P_{GAP}-CgCas9-T_{AOX1}	Lab stored
pEC1	pRSFDuet-P_{T7}-*AroG*	This study
pEC2	pRSFDuet-P_{T7}-*AroG*-P_{lac}-*AroB*	This study
pEC3	pRSFDuet-P_{T7}-*AroG*-P_{lac}-*AroB*-P_{tac}-*AroE*	This study
pTH1	pGAP-URA3-P_{GAP}-*XUT4*-T_{PDC1}-P_{PDC1}-*XI*-T_{ENO2}-P_{ENO2}-*XKS1*-T_{TDH3}-P_{TDH3}-*TKL1*-T_{TEF1}-P_{TEF1}-*TAL1*-T_{AOX1}	This study
pTH2	pGAP-HIS3-P_{GAP}-*FjTAL*-T_{PDC1}-P_{PDC1}-*HpaBC*-T_{AOX1}	This study
pTH3	pGAP-HIS3-P_{GAP}-*FjTAL*-T_{PDC1}-P_{PDC1}-*HpaBC*-T_{ENO2}-P_{ENO2}-*ARO4*^{K229L}-T_{TDH3}-P_{TDH3}-*ARO7*^{G141S}-T_{AOX1}	This study
pTH4	pGAP-TRP1-P_{GAP}-*MCH5*-T_{AOX1}	This study
pTH5	pGAP-TRP1-P_{GAP}-*RIB3*-T_{AOX1}	This study
pTH6	pGAP-TRP1-P_{GAP}-*RIB3*-T_{PDC1}-P_{PDC1}-*RIB4*-T_{AOX1}	This study
pTH7	pGAP-TRP1-P_{GAP}-*RIB3*-T_{PDC1}-P_{PDC1}-*RIB4*-T_{ENO2}-P_{ENO2}-*RIB5*-T_{AOX1}	This study
pTH8	pGAP-TRP1-P_{GAP}-*BsRIBBA*-T_{AOX1}	This study
pTH9	pGAP-TRP1-P_{GAP}-*BsRIBBA*-T_{PDC1}-P_{PDC1}-*FMN1*-T_{AOX1}	This study
pTH10	pGAP-TRP1-P_{GAP}-*BsRIBBA*-T_{PDC1}-P_{PDC1}-*FMN1*-T_{ENO2}-P_{ENO2}-*FAD1*-T_{AOX1}	This study
pTH11	pGAP-TRP1-P_{GAP}-*FLX1*-T_{AOX1}	This study
pTH12	pGAP-TRP1-P_{GAP}-*BsRIBBA*-T_{PDC1}-P_{PDC1}-*FLX1*-T_{AOX1}	This study
pTH13	pGAP-TRP1-P_{GAP}-*BsRIBBA*-T_{PDC1}-P_{PDC1}-*FLX1*-T_{ENO2}-P_{ENO2}-*MCH5*-T_{AOX1}	This study
pTH14	pGAP-TRP1-P_{GAP}-*BsRIBBA*-T_{PDC1}-P_{PDC1}-*FLX1*-T_{ENO2}-P_{ENO2}-*PDR5*-T_{AOX1}	This study
pTH15	pGAP-TRP1-P_{GAP}-*BsRIBBA*-T_{PDC1}-P_{PDC1}-*FLX1*-T_{ENO2}-P_{ENO2}-*PDR10*-T_{AOX1}	This study
pTH16	pGAP-TRP1-P_{GAP}-*BsRIBBA*-T_{PDC1}-P_{PDC1}-*FLX1*-T_{ENO2}-P_{ENO2}-*PDR11*-T_{AOX1}	This study
pTH17	pGAP-TRP1-P_{GAP}-*BsRIBBA*-T_{PDC1}-P_{PDC1}-*FLX1*-T_{ENO2}-P_{ENO2}-*PDR12*-T_{AOX1}	This study
pTH18	pGAP-TRP1-P_{GAP}-*BsRIBBA*-T_{PDC1}-P_{PDC1}-*FLX1*-T_{ENO2}-P_{ENO2}-*PDR15*-T_{AOX1}	This study
pTH19	pGAP-TRP1-P_{GAP}-*BsRIBBA*-T_{PDC1}-P_{PDC1}-*FLX1*-T_{ENO2}-P_{ENO2}-*SNQ2*-T_{AOX1}	This study
pTH20	pGAP-TRP1-P_{GAP}-*BsRIBBA*-T_{PDC1}-P_{PDC1}-*FLX1*-T_{ENO2}-P_{ENO2}-*ESBP6*-T_{AOX1}	This study
pTH21	pGAP-TRP1-P_{GAP}-*BsRIBBA*-T_{PDC1}-P_{PDC1}-*FLX1*-T_{ENO2}-P_{ENO2}-*AUS1*-T_{AOX1}	This study
pTH22	pGAP-TRP1-P_{GAP}-*BsRIBBA*-T_{PDC1}-P_{PDC1}-*FLX1*-T_{ENO2}-P_{ENO2}-*BPT1*-T_{AOX1}	This study
pTH23	pGAP-TRP1-P_{GAP}-*BsRIBBA*-T_{PDC1}-P_{PDC1}-*FLX1*-T_{ENO2}-P_{ENO2}-*YOR1*-T_{AOX1}	This study
pTH24	pGAP-TRP1-P_{GAP}-*BsRIBBA*-T_{PDC1}-P_{PDC1}-*FLX1*-T_{ENO2}-P_{ENO2}-*VMR1*-T_{AOX1}	This study
pTH25	pGAP-TRP1-P_{GAP}-*BsRIBBA*-T_{PDC1}-P_{PDC1}-*FLX1*-T_{ENO2}-P_{ENO2}-*NFT1*-T_{AOX1}	This study
pTH26	pGAP-TRP1-P_{GAP}-*BsRIBBA*-T_{PDC1}-P_{PDC1}-*FLX1*-T_{ENO2}-P_{ENO2}-*ESBP6*-T_{TDH3}-P_{TDH3}-*ARO1*-T_{TEF1}-P_{TEF1}-*ARO2*-T_{AOX1}	This study
pTH27	pGAP-HIS3-P_{GAP}-*ARO4*^{K229L}-T_{AOX1}	This study

pTH28	pGAP-HIS3-P_{GAP}-*ARO4*^{K229L}-T_{PDC1}-P_{PDC1}-*ARO1*-T_{AOX1}	This study

Table S3 Primers used in this study

Plasmids	Primers	Sequence (5'-3')
pEC1	AroG-F	GCCATCACCATCATCACCACATGAATTATCAGAACGACGA
	AroG-R	GCCGAGCTCGAATTCGGATCCTGGCTTTACCCGCGACGCGCTTTTA
pEC2	Lac-AroG-F	TAAAAGCGCGTCGCGGGTAAGCAAATATTATACGCAAGGC
	AroB-R	GCCGAGCTCGAATTCGGATCCTGGCTTTACGCTGATTGACAATCGG
pEC3	Tac-AroB-F	CCGATTGTCAATCAGCGTAAGGAGCTTATCGACTGCACGG
	AroE-R	GCCGAGCTCGAATTCGGATCCTGGCTTCACGCGGACAATTCCTCCTG
pTH1	XUT4-GAP-F	AACAAACACAATTACAAAAAATGTCTTCGTTATTGACTAAC
	XUT4-AOX1-R	CCGCATTCTTATCGCGGATCCGCGTTATTCCATCTCATTCAACTTGT
	PDC1-XUT4-F	AGTTGAATGAGATGGAATAATGGTATCCTCTATCGTATCGTATC
	XI-ENO2-R	TTGGGAGTGGATTCTCCCTGGAAATTATCTGAACAAGATGTTGTTG
	ENO2-XI-F	ACAACATCTTGTTCAGATAATTTCCAGGGAGAATCCACTC
	XKS1-TDH3-R	GCAGTATTGATAATGATAAACTCGAACTCTATAACGTTCTCTTTTTC
	TDH3-XKS1-F	GAAAAAGAGAACGTTATAGAGTTCGAGTTTATCATTATCAATACTGC
	TKL1-TEF1-R	TTTGAAGCTATGGTGTGTGGTCAGAAGGCAGAACGCAATGG
	TEF1-TKL1-F	CATTGCGTTCTGCCTTCTGACCACACACCATAGCTTCAAA
	TAL1-AOX1-R	CCGCATTCTTATCGCGGATCCGCGTTAGAATCTGGCTTCCAATTGTT
pTH2	FjTAL-GAP-F	AACAAACACAATTACAAAAAATGAATACCATCAATGAGTA
	FjTAL-AOX1-R	CGATACGATAGAGGATACCATTAATTATTAATCAAATGATCTT
	PDC1-FjTAL-F	TCATTTGATTAATAATTAATGGTATCCTCTATCGTATCGTATC
	HpaBC-AOX1-R	CCGCATTCTTATCGCGGATCCGCGTTAAACTGGAGCTTCCATTTCCA
pTH3	ENO2-HpaBC-F	AAATGGAAGCTCCAGTTTAATTTCCAGGGAGAATCCACTC
	$ARO4^{K229L}$-TDH3-R	ATTGATAATGATAAACTCGATTATTTCTTGTTAACTTCTCTTC
	TDH3-$ARO4^{K229L}$-F	GAGAAGTTAACAAGAAATAATCGAGTTTATCATTATCAATACTGC
	$ARO7^{G141S}$-AOX1-R	AACACACATAAACAAACAAAATGGATTTCACAAAACCAGA
pTH4	ENO2-HpaBC-F	AAATGGAAGCTCCAGTTTAATTTCCAGGGAGAATCCACTC
	$ARO4^{K229L}$-TDH3-R	ATTGATAATGATAAACTCGATTATTTCTTGTTAACTTCTCTTC
	TDH3-$ARO4^{K229L}$-F	GAGAAGTTAACAAGAAATAATCGAGTTTATCATTATCAATACTGC
	$ARO7^{G141S}$-AOX1-R	AACACACATAAACAAACAAAATGGATTTCACAAAACCAGA
pTH5	MCH5-GAP-F	AACAAACACAATTACAAAAAATGAGCTCAGACAGTTTAAC
	MCH5-AOX1-R	CCGCATTCTTATCGCGGATCCGCGTTAAAATCTGACCCACTTGAAG
pTH6	RIB3-GAP-F	AACAAACACAATTACAAAAAATGTTTACACCAATTGATCA
	RIB3-AOX1-R	CCGCATTCTTATCGCGGATCCGCGTTATTTCTTCAAATATTGGG
pTH7	PDC1-RIB3-F	CCCAATATTTGAAGAAATAATGGTATCCTCTATCGTATCGTATC
	RIB4-AOX1-R	CCGCATTCTTATCGCGGATCCTCAAAAAGCATTTTTACCGA
pTH8	ENO2-RIB4-F	TCGGTAAAAATGCTTTTTGATTTCCAGGGAGAATCCACTC
	RIB5-AOX1-R	CCGCATTCTTATCGCGGATCCGCGTTATTTATTTAGGTAGTTTC
pTH9	BsRIBBA-GAP-F	AACAAACACAATTACAAAAAATGTTTCATCCGATAGAAGA
	BsRIBBA-AOX1-R	CCGCATTCTTATCGCGGATCCGCGTTAGAAATGAAGTAAATGAC
pTH10	PDC1-BsRIBBA-F	GTCATTTACTTCATTTCTAATGGTATCCTCTATCGTATCGTATC
	FMN1-AOX1-R	CCGCATTCTTATCGCGGATCCCTATAATTGTTGTTTGAATA
pTH11	ENO2-FMN1-F	TATTCAAACAACAATTATAGTTTCCAGGGAGAATCCACTC
	FAD1-AOX1-R	CCGCATTCTTATCGCGGATCCGCGTTAATTCTTGATCCTGCCTG

pTH12	FLX1-GAP-F	AACAAACACAATTACAAAAAATGGTCGATCACCAGTGGA
	FLX1-AOX1-R	CCGCATTCTTATCGCGGATCCGCGCTAAAGCCTATGCTTAAGGTTC
pTH13	PDC1-BsRIBBA-F	GTCATTTACTTCATTTCTAATGGTATCCTCTATCGTATCGTATC
	FLX1-AOX1-R	CCGCATTCTTATCGCGGATCCGCGCTAAAGCCTATGCTTAAGGTTC
pTH14	TDH3-FAD1-F	CAGGCAGGATCAAGAATTAATCGAGTTTATCATTATCAATACTGC
	FLX1-AOX1-R	CCGCATTCTTATCGCGGATCCGCGCTAAAGCCTATGCTTAAGGTTC
pTH15	ENO2-FLX1-F	ACCTTAAGCATAGGCTTTAGTTTCCAGGGAGAATCCACTC
	PDR5-AOX1-R	CCGCATTCTTATCGCGGATCCGCGTTATTTCTTGGAGAGTTTACCGT
pTH16	ENO2-FLX1-F	ACCTTAAGCATAGGCTTTAGTTTCCAGGGAGAATCCACTC
	PDR10-AOX1-R	CCGCATTCTTATCGCGGATCCGCGTTATTTCTTTAATTTTTTGCTTTTC
pTH17	ENO2-FLX1-F	ACCTTAAGCATAGGCTTTAGTTTCCAGGGAGAATCCACTC
	PDR11-AOX1-	CCGCATTCTTATCGCGGATCCGCGTTATACGCTTTGTTCGTTTGGA
pTH18	ENO2-FLX1-F	ACCTTAAGCATAGGCTTTAGTTTCCAGGGAGAATCCACTC
	PDR12-AOX1-R	CCGCATTCTTATCGCGGATCCGCGTTATTTCTTCGTGATTTTATTTTCG
pTH19	ENO2-FLX1-F	ACCTTAAGCATAGGCTTTAGTTTCCAGGGAGAATCCACTC
	PDR15-AOX1-R	CCGCATTCTTATCGCGGATCCGCGTCACTTCTTGGGTTTTTCGGAAA
pTH20	ENO2-FLX1-F	ACCTTAAGCATAGGCTTTAGTTTCCAGGGAGAATCCACTC
	SNQ2-AOX1-R	CCGCATTCTTATCGCGGATCCGCGTTACTGCTTCTTTTTCCTTATGT
pTH21	ENO2-FLX1-F	ACCTTAAGCATAGGCTTTAGTTTCCAGGGAGAATCCACTC
	ESBP6-AOX1-R	CCGCATTCTTATCGCGGATCCGCGCTAGACCTTCATTGGATATACCA
pTH22	ENO2-FLX1-F	ACCTTAAGCATAGGCTTTAGTTTCCAGGGAGAATCCACTC
	AUS1-AOX1-R	CCGCATTCTTATCGCGGATCCGCGTTAGTTCTGTACAGGCTTCTTCC
pTH23	ENO2-FLX1-F	ACCTTAAGCATAGGCTTTAGTTTCCAGGGAGAATCCACTC
	BPT1-AOX1-R	CCGCATTCTTATCGCGGATCCGCGTTATTTCAAATACCCACCTTTC
pTH24	ENO2-FLX1-F	ACCTTAAGCATAGGCTTTAGTTTCCAGGGAGAATCCACTC
	YOR1-AOX1-R	CCGCATTCTTATCGCGGATCCGCGTTAACTTCTGTTCTCGAAATCA
pTH25	ENO2-FLX1-F	ACCTTAAGCATAGGCTTTAGTTTCCAGGGAGAATCCACTC
	VMR1-AOX1-R	CCGCATTCTTATCGCGGATCCGCGTTATTTCATCATCTTACTTGATTG
pTH26	ENO2-FLX1-F	ACCTTAAGCATAGGCTTTAGTTTCCAGGGAGAATCCACTC
	NFT1-AOX1-R	CCGCATTCTTATCGCGGATCCGCGTTATCTTTTATTATCGAATGAGA
pTH27	TDH3-ESBP6-F	TATATCCAATGAAGGTCTAGTCGAGTTTATCATTATCAATACTGC
	ARO1-TEF1-R	TTTGAAGCTATGGTGTGTGGCTACTCAGAAGTGACTGCCTC
	TEF1-ARO1-F	AGGCAGTCACTTCTGAGTAGCCACACACCATAGCTTCAAA
	ARO2-AOX1-R	CCGCATTCTTATCGCGGATCCGCGTTAATAGACAGCATTAAATGAGGA
pTH28	ARO4^{K229L}-GAP-F	AACAAACACAATTACAAAAAATGAGTGAATCTCCAATGTT
	ARO4^{K229L}-AOX1-R	CCGCATTCTTATCGCGGATCCGCGTTATTTCTTGTTAACTTCTCTT
pTH29	PDC1-ARO4^{K229L}-F	GAGAAGTTAACAAGAAATAATGGTATCCTCTATCGTATCGTATC
	ARO1-PDC1-R	ACACAGCAAAACACAAAAATATGGTTCAAGAAGGGCATGT
	ARO1-AOX1-R	CCGCATTCTTATCGCGGATCCGCGCTACTCAGAAGTGACTGCCTCGA
Cas9 and SgRNA	GAP-F	CACCACAGCAGCACCAAC
	AOX1-R	TCTCACTTAATCTTCTGTACTCTGAAGAG
Dornor	HIS3-F	catagagaagagaatctttatctatctcacagaaaagagaatctacgaccCACCACAGCAGCACCAAC
	HIS3-R	gtttttttttctttttcgatagtgtatattaaggtgtagaagaatagtgATACGCGGAACAATCAAT
	PHA2-F	ggagagagaatgatctctaggagaagcataatcaacatcaaacgcggacaCACCACAGCAGCACCAAC

PHA2-R	acgagtctacccaaaaaaaaaaaaaacaaaaaaaaaaaggactcactctaGTGGTGCTTTTACAATGCGG
LYS2-F	cctgctaattcatcttcatcactcagttttgtccatcttaacaatagacaCACCACAGCAGCACCAAC
LYS2-R	atatatgtatgtatgtatataattgtattcaagttaagccgtagaactcaCGATTTGCTCTTCGATGCCA
ARO2-F	atgtccacatttggtactatcttccgtgtcactacatacggtgaatcccaCACCACAGCAGCACCAAC
ARO2-R	ctaatagacagcattaaatgaggattctcttgatttctgaatcaagtaagCTACTCAGAAGTGACTGCCTC

Table S4 Composition of sugarcane bagasse hydrolysate

**Composition**	**Bagasse hydrolysate (g/L)**
Glucose (C6)	42.4
Xylose (C5)	15.2
5-hydroxymethylfurfural	0.22
furfural	1.36
formic acid	0.12
acetic acid	2.78
